# Formation of TiB_2_–MgAl_2_O_4_ Composites by SHS Metallurgy

**DOI:** 10.3390/ma16041615

**Published:** 2023-02-15

**Authors:** Chun-Liang Yeh, Fu-You Zheng

**Affiliations:** Department of Aerospace and Systems Engineering, Feng Chia University, Taichung 40724, Taiwan

**Keywords:** MgAl_2_O_4_, TiB_2_, metallothermic reduction, SHS powder metallurgy, combustion wave propagation

## Abstract

TiB_2_–MgAl_2_O_4_ composites were fabricated by combustion synthesis involving metallothermic reduction reactions. Thermite reagents contained Al and Mg as dual reductants and TiO_2_ or B_2_O_3_ as the oxidant. The reactant mixtures also comprised elemental Ti and boron, as well as a small amount of Al_2_O_3_ or MgO to serve as the combustion moderator. Four reaction systems were conducted and all of them were exothermic enough to proceed in the mode of self-propagating high-temperature synthesis (SHS). The reaction based on B_2_O_3_/Al/Mg thermite and diluted with MgO was the most exothermic, while that containing TiO_2_/Al/Mg thermite and Al_2_O_3_ as the diluent was the least. Depending on different thermites and diluents, the combustion front temperatures in a range from 1320 to 1720 °C, and combustion wave velocity from 3.9 to 5.7 mm/s were measured. The XRD spectra confirmed in situ formation of TiB_2_ and MgAl_2_O_4_. It is believed that MgAl_2_O_4_ was synthesized through a combination reaction between Al_2_O_3_ and MgO, both of which can be totally or partially produced from the metallothermic reduction of B_2_O_3_ or TiO_2_. The microstructure of the TiB_2_–MgAl_2_O_4_ composite exhibited fine TiB_2_ crystals surrounded by large densified MgAl_2_O_4_ grains. This study demonstrated an energy-saving and efficient route for fabricating MgAl_2_O_4_-containing composites.

## 1. Introduction

TiB_2_ has been one of the most studied ultra-high temperature ceramics (UHTCs) due to its unique properties, including a high melting point (3225 °C), high hardness (33 MPa), high Young’s modulus (530 MPa), excellent wear and oxidation resistance, thermal shock resistance, chemical inertness, and good electric conductivity [1,2,3]. Combination of these properties makes TiB_2_ an ideal candidate for use in ballistic armors, crucibles, metal evaporation boats, cutting tools, wear resistance parts, and cathodes for alumina smelting [4,5,6]. Many ceramic phases, such as Al_2_O_3_, SiC, B_4_C, and MgAl_2_O_4_, have been considered as the reinforcement to improve fracture toughness, oxidation resistance, heat resistance, and mechanical strength of the TiB_2_-based composites [7,8,9,10]. Moreover, a recent study showed that TiB_2_–Al_2_O_3_–MgAl_2_O_4_ composite possesses temperature insensitive and enhanced microwave absorption properties [11]. Magnesium aluminate spinel, MgAl_2_O_4_, as an additive has been rarely studied, possibly because the fabrication of MgAl_2_O_4_ via either the direct solid-state reaction of oxides or wet chemical methods requires multiple steps that are complicated and time-consuming [12,13,14]. However, MgAl_2_O_4_ is an attractive component due to its high melting point, chemical inertness, high hardness, corrosion resistance, high mechanical strength, and low cost [12]. 

Among various fabrication routes for preparing multiphasic ceramics, metallothermic reduction reactions (i.e., thermite reactions) combined with combustion synthesis have been recognized as a promising technique for in situ formation of MgAl_2_O_4_-containing composites [15]. Combustion synthesis in the mode of self-propagating high-temperature synthesis (SHS), which is based on strongly exothermic reactions, has merits of low energy consumption, short reaction time, simple equipment and operation, high-purity products, and in situ formation of composite components [16,17,18]. Moreover, aluminothermic and magnesiothermic reduction reactions have Al_2_O_3_ and MgO as their respective by-products, both of which are precursors for the formation of MgAl_2_O_4_. Consequently, Omran et al. [19] applied the reduction-based SHS technique to produce MgAl_2_O_4_–W–W_2_B composites through magnesiothermic reduction of B_2_O_3_ and WO_3_ in the presence of Al_2_O_3_. Zaki et al. [20] synthesized MoSi_2_– and Mo_5_Si_3_–MgAl_2_O_4_ composites by SHS with a reducing stage from raw materials consisting of MoO_3_, SiO_2_, and Al as aluminothermic reagents and MgO as a precursor. Similarly, MgO was added into the reactive mixture of TiO_2_, B_2_O_3_, and Al to fabricate TiB_2_–MgAl_2_O_4_ composites by thermitic combustion synthesis [21]. Generally, most of the previous studies on the formation of MgAl_2_O_4_-containing composites had prior addition of one of two precursors (Al_2_O_3_ or MgO) in the green samples. 

This study represents the first attempt to prepare TiB_2_–MgAl_2_O_4_ composites from the SHS powder metallurgy simultaneously involving aluminothermic and magnesiothermic reduction of TiO_2_ or B_2_O_3_. Only a small amount of Al_2_O_3_ or MgO was included in the reactive mixture to serve as the combustion moderator and part of the precursors for the formation of MgAl_2_O_4_. Four SHS reaction systems formulated with different metallothermic reagents and combustion diluents were investigated. In this work, combustion exothermicity and kinetics of the combustion wave of the SHS process, as well as compositions and microstructures of the final products were explored. 

## 2. Materials and Methods

The starting materials adopted by this study included TiO_2_ (Acros Organics, Geel, Belgium, 99.5%), B_2_O_3_ (Acros Organics, 99%), Al_2_O_3_ (Alfa Aesar, Haverhill, MA, USA, 99%), MgO (Acros Organics, 99.5%), Al (Showa Chemical Co., Tokyo, Japan, <45 μm, 99.9%), Mg (Alfa Aesar, <45 μm, 99.8%), Ti (Alfa Aesar, <45 μm, 99.8%), and amorphous boron (Noah Technologies, San Antonio, TX, USA, <1 μm, 93.5%). Four SHS reactions were formulated for the synthesis of 3TiB_2_–MgAl_2_O_4_ composites. Two metallothermic reagents (i.e., thermites) were considered; one is composed of TiO_2_, Al, and Mg, as shown in Equations (1) and (2), and the other comprises B_2_O_3_, Al, and Mg, as in Equations (3) and (4). Due to strong exothermicity of combustion, Al_2_O_3_ with an amount of 0.3 mol. was included in Equations (1) and (3) as the combustion moderator (or combustion diluent) in order to attain stable propagation of the combustion wave. The pre-added Al_2_O_3_ also acted as part of the precursor for the synthesis of MgAl_2_O_3_. Likewise, an equal amount of MgO was adopted by Equations (2) and (4) and MgO played the same role as Al_2_O_3_ in Equations (1) and (3).

(1) $$\left(1.55TiO_{2}+1.4Al+Mg\right)+0.3Al_{2}O_{3}+1.45Ti+6B\to 3TiB_{2}+MgAl_{2}O_{4}$$

(2) $$\left(1.85TiO_{2}+2Al+0.7Mg\right)+0.3MgO+1.15Ti+6B\to 3TiB_{2}+MgAl_{2}O_{4}$$

(3) $$\left(1.033B_{2}O_{3}+1.4Al+Mg\right)+0.3Al_{2}O_{3}+3Ti+3.934B\to 3TiB_{2}+MgAl_{2}O_{4}$$

(4) $$\left(1.233B_{2}O_{3}+2Al+0.7Mg\right)+0.3MgO+3Ti+3.534B\to 3TiB_{2}+MgAl_{2}O_{4}$$

Combustion exothermicity of the above four reactions, Equations (1)–(4), was evaluated by calculating their adiabatic combustion temperatures (*T*_ad_) from the following energy balance equation [17,22] with thermochemical data taken from [23].

$$\Delta H_{r}+\overset{T_{ad}}{\underset{298}{\int }}\sum n_{j}c_{p}\left(P_{j}\right)dT+\underset{298-T_{ad}}{\sum }n_{j}L\left(P_{j}\right)=0$$

where ∆*H_r_* is the reaction enthalpy at 298 K, *c_p_* and *L* are the heat capacity and latent heat, *n_j_* is the stoichiometric coefficient, and *P_j_* refers to the product component. 

The SHS experiments were conducted in a windowed combustion chamber filled with Ar at 0.25 MPa. Reactant powders were well mixed in a tubular ball mill and then uniaxially compressed into cylindrical test specimens with a diameter of 7 mm, a height of 12 mm, and a relative density of 55%. The sample compact was ignited by an electrically heated tungsten coil. An R-type bare-wire thermocouple (Pt/Pt-13%Rh) with a bead size of 125 μm was used to measure the combustion temperature. The propagation velocity of combustion wave (*V*_f_) was determined by the time derivative of the flame-front trajectory constructed from the recorded series of combustion images. Phase compositions of the products were identified by an X-ray diffractometer (XRD, Bruker D2 Phaser, Karlsruhe, Germany). Microstructures and constituent elements of the products were examined by the scanning electron microscopy (SEM, Hitachi, Tokyo, Japan, S3000H) and energy dispersive spectroscopy (EDS). Details of the experimental methods were reported elsewhere [24]. 

## 3. Results and Discussion

### 3.1. Combustion Exothermicity of Reduction-Based SHS Reactions

**Figure 1:**
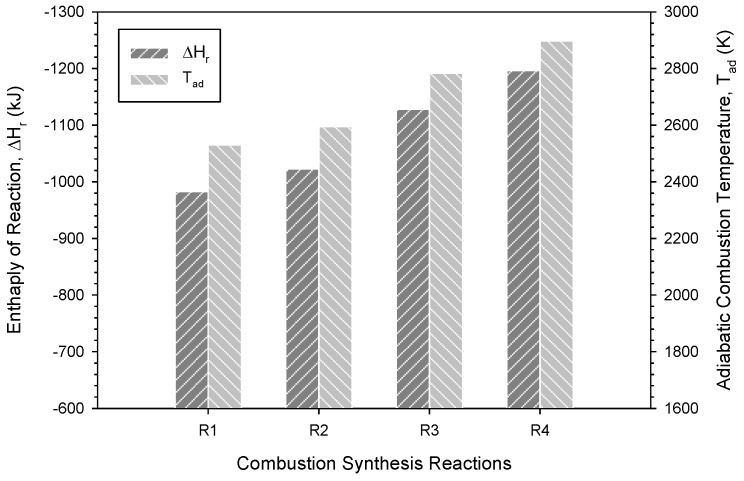
Enthalpies of reaction (∆*H*_r_) and adiabatic combustion temperatures (*T*_ad_) of Equation (1)–(4) for the synthesis of 3TiB_2_–MgAl_2_O_4_ composites.

Figure 1 presents the calculated ∆*H*_r_ and *T*_ad_ of reactions Equations (1)–(4) and shows that Equation (4) has the highest values while Equation (1) has the lowest ones. Both ∆*H*_r_ and *T*_ad_ increase from Equations (1)–(4). Specifically, the values of *T*_ad_ are 2530 K, 2595 K, 2783 K, 2897 K for Equations (1)–(4), respectively. A comparison between Equations (1) and (2) revealed that the combustion moderator Al_2_O_3_ appeared to impose a stronger dilution effect on combustion than MgO, which led to a lower *T*_ad_ for Equation (1) than Equation (2). Similar results were observed in Equations (3) and (4). These findings could also be explained by the fact that metallothemic reduction of TiO_2_ or B_2_O_3_ by Al is more exothermic than that by Mg [15,25]. 

On the other hand, because the B_2_O_3_-based thermite is more energetic than the one using TiO_2_ [25], Equation (3) has a higher *T*_ad_ than Equation (1). Similarly, Equation (4) has a higher *T*_ad_ than Equation (2). According to the calculated *T*_ad_, it is realized that the thermite oxidants (i.e., B_2_O_3_ versus TiO_2_) have a more pronounced influence on combustion exothermicity than the diluent oxides (i.e., Al_2_O_3_ versus MgO). 

### 3.2. Combustion Temperature and Self-Propagating Velocity

**Figure 2:**
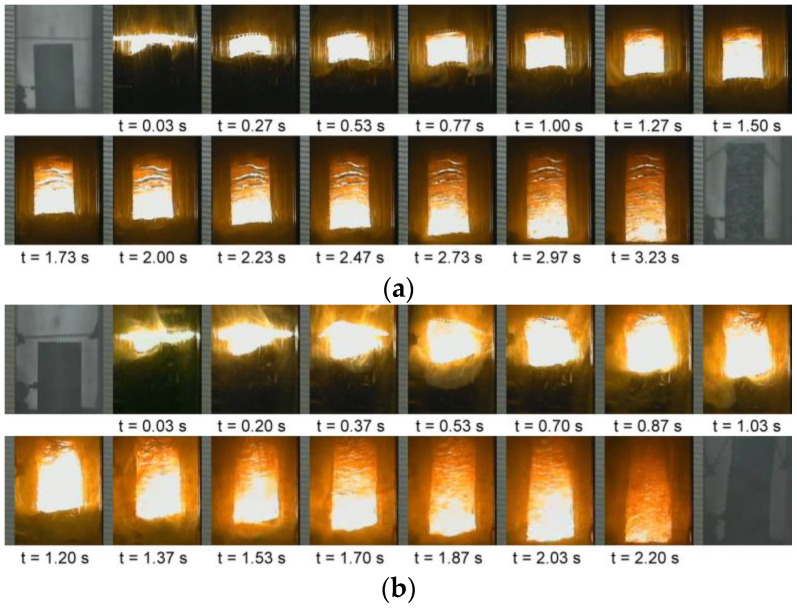
Time sequences of recorded SHS images illustrating self-sustaining combustion wave of (**a**) Equation (1) and (**b**) Equation (3).

Two series of the SHS processes recorded from reactions Equations (1) and (3) are illustrated in Figure 2a,b, respectively. It is apparent that upon ignition, the reaction was initiated and characterized by a self-sustaining combustion wave. More intense combustion accompanied with a faster combustion wave was observed in Figure 2b, when compared with that in Figure 2a. Combustion luminosity and flame spreading speed reflected the degree of reaction exothermicity. As mentioned above, B_2_O_3_/Al/Mg-based Equation (3) is more energetic than TiO_2_/Al/Mg-based Equation (1). Similar combustion behavior was also noticed in Equations (2) and (4).

**Figure 3:**
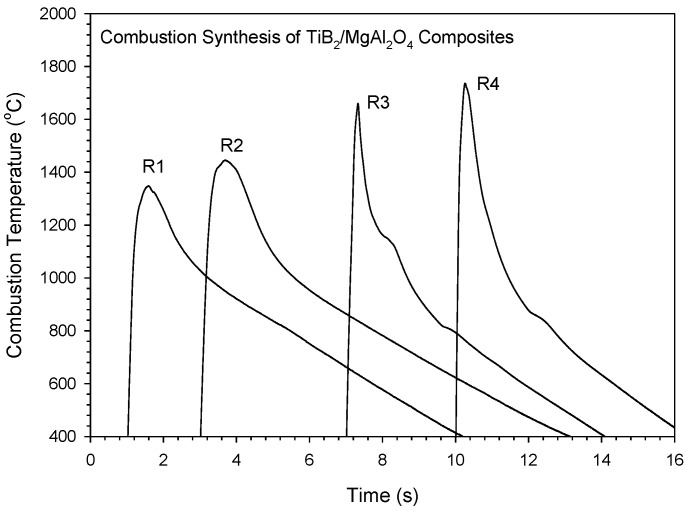
Typical combustion temperature profiles measured from Equation (1)–(4) for the synthesis of 3TiB_2_–MgAl_2_O_4_ composites.

Figure 3 depicts typical combustion temperature profiles measured from four different reactions. All profiles exhibit a steep temperature rise followed by a rapid descent, which is characteristic of the SHS reaction that features a fast combustion wave and a thin reaction zone. The peak value is considered as the combustion front temperature (*T*_c_). When compared with pinnacles in the contours of Equations (1) and (2), sharper peaks were detected in the profiles of Equations (3) and (4). This implied a faster combustion wave in Equations (3) and (4). As shown in Figure 3, the values of *T*_c_ from Equation (1)–(4) in ascending order are 1348 °C, 1445 °C, 1660 °C, and 1736 °C. It should be noted that the measured combustion front temperatures are in agreement with the calculated reaction exothermicity.

It is useful to note in Figure 3 that the curves of Equations (3) and (4) have a shape peak with a pronounced shoulder. The shape peak was a result of the fast combustion wave. The pronounced shoulder could be caused by the occurrence of volumetric synthesis reactions after the passage of the rapid combustion wave. 

**Figure 4:**
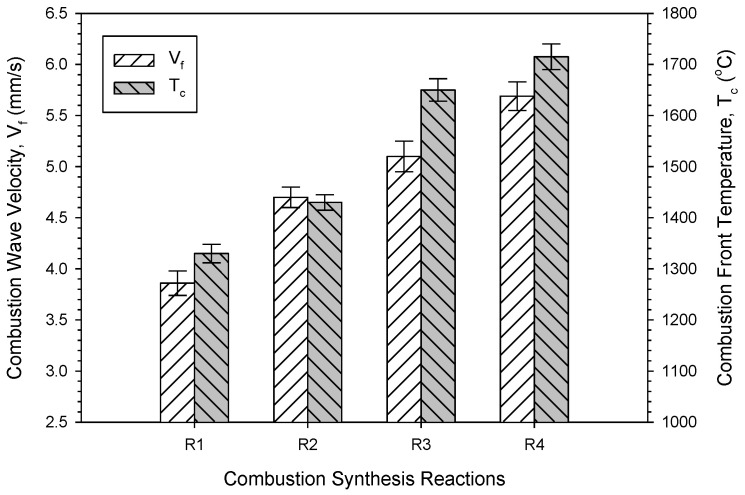
Combustion wave velocity (*V*_f_) and combustion front temperature (*T*_c_) measured from Equation (1)–(4) for the synthesis of 3TiB_2_–MgAl_2_O_4_ composites.

Figure 4 plots the measured combustion wave propagation velocities (*V*_f_) and temperatures (*T*_c_) of four reactions. The rising trend of *V*_f_ from Equations (1)–(4) is consistent with that of *T*_c_. This can be understood by the fact that the propagation of combustion wave is essentially governed by layer-by-layer heat transfer from the reaction zone to unreacted region, and therefore, is subject to the combustion front temperature. As presented in Figure 4, the average combustion velocities are 3.9, 4.7, 5.1, and 5.7 mm/s for Equation (1)–(4), respectively. It is worth noting that the measured combustion temperature not only justified the reaction exothermic analysis, but confirmed the temperature dependence of combustion wave velocity. 

### 3.3. Composition and Microstructure Analyses of Synthesized Products

**Figure 5:**
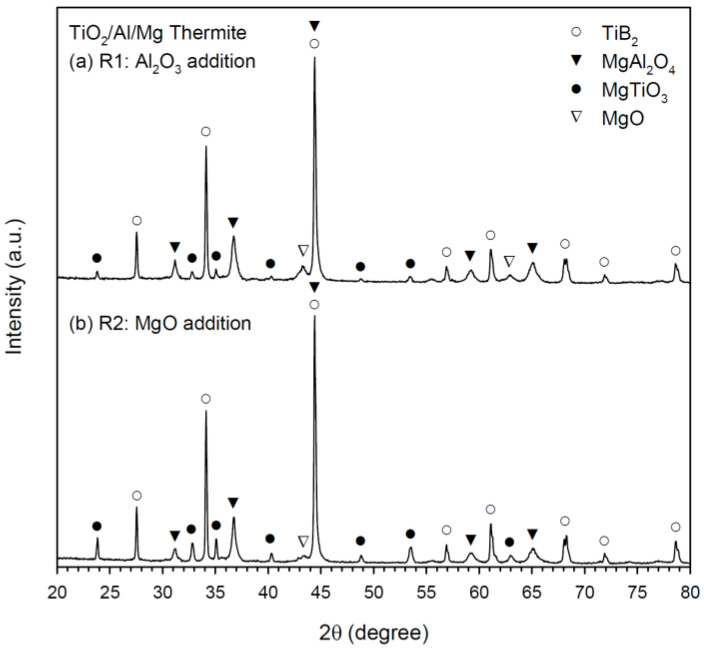
XRD patterns of TiB_2_/MgAl_2_O_4_ composites obtained from SHS reactions of (**a**) Equation (1) and (**b**) Equation (2).

The XRD spectra of the final products synthesized from Equations (1) and (2) are shown in Figure 5a,b, respectively. Both indicated the formation of TiB_2_ and MgAl_2_O_4_ along with two minor phases, magnesium titanate (MgTiO_3_) and MgO. It is believed that MgAl_2_O_4_ was synthesized through a combination reaction between Al_2_O_3_ and MgO. Equation (1) and (2) were formulated with the same thermite reagents of TiO_2_, Al, and Mg, but diluted by different metal oxides. That is, Al_2_O_3_ was partly pre-added and partly thermite-produced, while MgO was completely generated from the reduction of TiO_2_ by Mg in Equation (1). In contrast, the required Al_2_O_3_ in Equation (2) was entirely produced from the reduction of TiO_2_ by Al, but MgO was supplied in part from prior addition and in part from the reduction of TiO_2_ by Mg. For both Equations (1) and (2), TiB_2_ was synthesized from the reaction of elemental boron with reduced and metallic Ti. 

Traces of MgO suggested an incomplete reaction due probably to the relatively low reaction temperatures of Equations (1) and (2). The presence of MgTiO_3_ in the final products of Equations (1) and (2) could be attributed to the reaction of MgO with the thermite oxidant TiO_2_ [26,27]. The formation of MgTiO_3_ in the SHS-produced TiB_2_–MgAl_2_O_4_ composites was also observed by Radishevskaya et al. [10] using Ti, boron, and MgAl_2_O_4_ as their starting materials and a partial decomposition of MgAl_2_O_4_ during combustion synthesis was considered as a possible route resulting in the formation of MgTiO_3_. 

The presence of MgO along with no detection of Al_2_O_3_ in the final products of Equations (1) and (2) suggested that the as-synthesized MgAl_2_O_4_ is an Al_2_O_3_-rich spinel. The formation of MgTiO_3_ could also result in the production of Al_2_O_3_-rich spinel. According to Naghizadeh et al. [28], magnesium titanate compounds (MgTiO_3_ and Mg_2_TiO_4_) were identified in the phase evolution of MgAl_2_O_4_ produced from TiO_2_-containing samples and stoichiometric MgAl_2_O_4_ spinel shifted toward the Al_2_O_3_-rich type. Due to the formation of MgTiO_3_, the amount of TiB_2_ formed in the composite should be less than the stoichiometric amount. 

**Figure 6:**
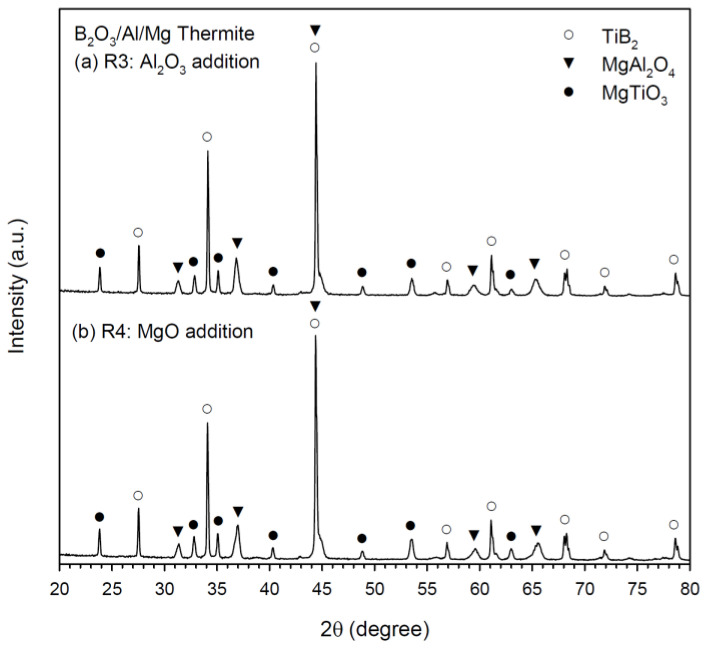
XRD patterns of TiB_2_/MgAl_2_O_4_ composites obtained from SHS reactions of (**a**) Equation (3) and (**b**) Equation (4).

Figure 6a,b exhibits the XRD patterns of the synthesized composites from Equations (3) and (4), respectively. In addition to TiB_2_ and MgAl_2_O_4_, a small amount of MgTiO_3_ was identified. The formation of MgAl_2_O_4_ from a combination reaction between Al_2_O_3_ and MgO was proved. Both Al_2_O_3_ and MgO can be totally or partially produced from the reduction of B_2_O_3_ by Al and Mg. For Equations (3) and (4) containing B_2_O_3_/Al/Mg-based thermite, TiB_2_ was produced from the reaction of metallic Ti with reduced and elemental boron. Moreover, the formation of MgTiO_3_ in Equations (3) and (4) might involve some interaction of Ti with B_2_O_3_ to form TiO_2_ which further reacted with MgO. Unlike that in Equations (1) and (2), MgO was no longer detected in the final products of Equations (3) and (4). 

MgTiO_3_ ceramic has been proved to be an excellent dielectric material, owing to its high dielectric constant, low dielectric loss, high value of quality factors, and good temperature stability [29,30]. It is believed that a trace amount of MgTiO_3_ as a minor phase existed in the as-synthesized TiB_2_-MgAl_2_O_4_ products has no effect on the refractory properties of the composites. However, removal of MgTiO_3_ from the TiB_2_-MgAl_2_O_4_ composite would be difficult, since it could combine with MgAl_2_O_4_ in a solid solution form [28]. 

**Figure 7:**
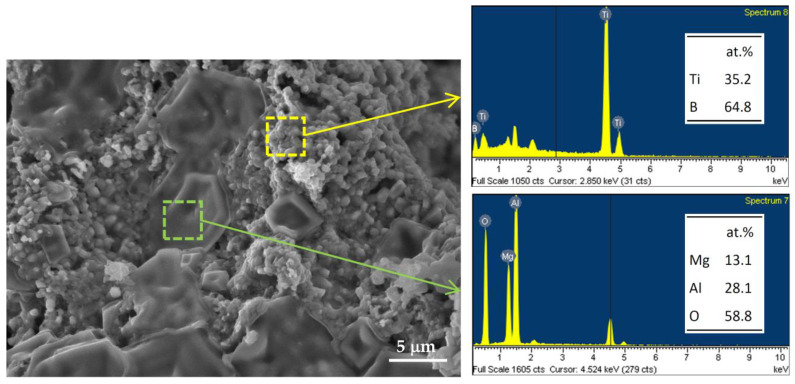
SEM image and EDS spectra of TiB_2_/MgAl_2_O_4_ composite obtained from the SHS reaction of Equation (1).

The SEM image shown in Figure 7 illustrates the microstructure of fracture surface of the product synthesized from Equation (1) which contains a TiO_2_/Al/Mg-based thermite. The morphology displays several large and solidified MgAl_2_O_4_ aggregates of 5–15 μm surrounded by fine-grain TiB_2_ crystals with a particle size of about 1–2 μm. Moreover, EDS analysis of two characteristic regions in the product surface indicates that the atomic ratios of Ti:B = 35.2:64.8 and Mg:Al:O = 13.1:28.1:58.8 match well with the stoichiometries of TiB_2_ and MgAl_2_O_4_, respectively. 

**Figure 8:**
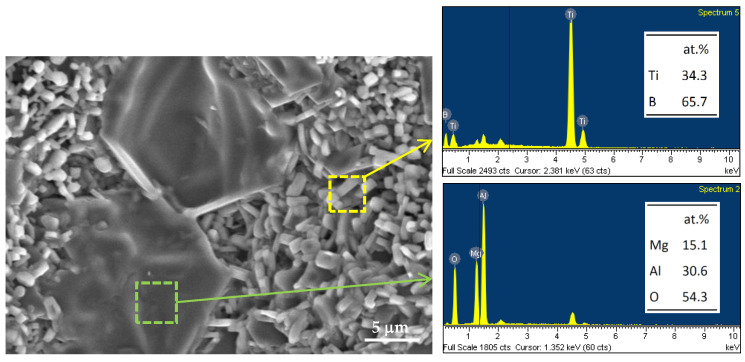
SEM image and EDS spectra of TiB_2_/MgAl_2_O_4_ composite obtained from the SHS reaction of Equation (4).

For the final product of B_2_O_3_/Al/Mg-based Equation (4), the microstructure and elemental ratios of the components are presented in Figure 8. As can be seen, MgAl_2_O_4_ was formed as large densified aggregates of around 20 μm and TiB_2_ crystals were in a short-rod form with a length of 2–4 μm or in a shape of fine grains of 1–2 μm. Based on the EDS analysis, the atomic ratio of the selected area in an aggregate is Mg:Al:O = 15.1:30.6:54.3 that is reasonably close to MgAl_2_O_4_. Short-rod crystals have a composition of Ti:B = 34.3:65.7, which certainly is TiB_2_. 

In summary, the addition of MgAl_2_O_4_ into TiB_2_ enhanced the refractory properties, such as the high-temperature oxidation and corrosion resistance and thermal shock resistance [10,31,32]. Like many sintering aids, MgAl_2_O_4_ as an additive could improve densification of TiB_2_ ceramics and reduce sintering temperatures [33,34]. The abnormal grain growth could be efficiently prevented during the sintering process. As a result, it is more likely to obtain a uniform grain distribution. 

Moreover, the TiB_2_-MgAl_2_O_4_ composite is a promising high-temperature microwave absorption material with a reflection loss less than –5 dB at 8.2–18.0 GHz in the temperature range of 25 °C to 1100 °C [11]. The composite also exhibits an extremely high tolerance against intense irradiation in harsh environments [35,36]. Therefore, the potential uses of the TiB_2_-MgAl_2_O_4_ composite might include heat-resistant coatings, nozzles and nose cones of supersonic jets, microwave absorption components, diagnostic or detector windows in fusion devices, target materials in the nuclear applications, etc., [11,35,36]. 

## 4. Conclusions

In situ formation of 3TiB_2_–MgAl_2_O_4_ composites was conducted by combustion synthesis combined with metallothermic reduction reactions involving Al and Mg as dual reductants. Thermite reagents with different oxidants were considered; one utilized TiO_2_ and the other B_2_O_3_. The reactant mixtures also contained elemental Ti and boron. This study in total completed four SHS reactions, within which a small amount of Al_2_O_3_ or MgO was included in the reactive mixture to serve as the combustion moderator and part of the precursors for the formation of MgAl_2_O_4_. The overall synthesis reaction was exothermic enough to proceed in the SHS mode. An energy-saving and efficient fabrication route for the formation MgAl_2_O_4_-containing composites was demonstrated. 

The analysis of combustion exothermicity indicated that the SHS reaction containing B_2_O_3_/Al/Mg-based thermites was more energetic than that adopting TiO_2_ as the oxidant. Prior addition of Al_2_O_3_ had a greater cooling effect on combustion than that of MgO. Depending on different thermites and diluents, the measured combustion front temperatures ranged from 1320 to 1720 °C, and combustion wave velocity from 3.9 to 5.7 mm/s. The temperature dependence of combustion wave velocity was justified. The XRD analysis confirmed in situ formation of TiB_2_ and MgAl_2_O_4_. A small amount of MgTiO_3_ was found as the impurity. It is believed that MgAl_2_O_4_ was synthesized through a combination reaction between Al_2_O_3_ and MgO, both of which can be totally or partially produced from the metallothermic reduction of B_2_O_3_ or TiO_2_. The microstructure of the synthesized composite exhibited that MgAl_2_O_4_ was surrounded by closely packed TiB_2_ grains. MgAl_2_O_4_ was formed as densified aggregates with a size of 5–20 μm. TiB_2_ crystals were produced in a shape of short rods of 2–4 μm and fine grains of 1–2 μm. 

## Data Availability

Data presented in this study are available in the article.
